# Mast Cells in Cardiovascular Disease: From Bench to Bedside

**DOI:** 10.3390/ijms20143395

**Published:** 2019-07-10

**Authors:** M. A. W. Hermans, J. E. Roeters van Lennep, P. L. A. van Daele, I. Bot

**Affiliations:** 1 Department of Internal Medicine, section of Clinical Immunology, Erasmus MC University Center, 3015 GD Rotterdam, The Netherlands; 2 Department of Internal Medicine, section of Vascular Medicine, Erasmus MC Erasmus MC University Center, 3015 GD Rotterdam, The Netherlands; 3 Division of BioTherapeutics, Leiden Academic Centre for Drug Research, Leiden University, 2300 RA Leiden, The Netherlands

**Keywords:** mast cell, cardiovascular, atherosclerosis, myocardial infarction, mastocytosis, Kounis syndrome, allergy, asthma

## Abstract

Mast cells are pluripotent leukocytes that reside in the mucosa and connective tissue. Recent studies show an increased prevalence of cardiovascular disease among patients with mastocytosis, which is a hematological disease that is characterized by the accumulation of mast cells due to clonal proliferation. This association suggests an important role for mast cells in cardiovascular disease. Indeed, the evidence establishing the contribution of mast cells to the development and progression of atherosclerosis is continually increasing. Mast cells may contribute to plaque formation by stimulating the formation of foam cells and causing a pro-inflammatory micro-environment. In addition, these cells are able to promote plaque instability by neo-vessel formation and also by inducing intraplaque hemorrhage. Furthermore, mast cells appear to stimulate the formation of fibrosis after a cardiac infarction. In this review, the available data on the role of mast cells in cardiovascular disease are summarized, containing both in vitro research and animal studies, followed by a discussion of human data on the association between cardiovascular morbidity and diseases in which mast cells are important: Kounis syndrome, mastocytosis and allergy.

## 1. Introduction

Mast cells (MC) are long-lived cells that reside in the connective tissue. They are part of the innate immune system and have pleiotropic functions and phenotypes. MC are strategically situated in the areas of the body that form a barrier to the outside world, such as the skin, the mucosal tissues and the airways [1]. Furthermore, low numbers of these MC are present in the perivascular tissue of large blood vessels and between, e.g., the cardiomyocytes in heart tissue. MC precursors arise from hematopoietic stem cells in bone marrow. The route of development towards mature MCs is not entirely clear yet, but it is assumed that MC precursors are formed from myeloid progenitor cells [2]. MC precursors leave the bone marrow to mature in tissue. The exact MC phenotype in tissue is controlled by their location and their direct environment. Roughly, two subtypes of MC are recognized: MC_TC_ that produce the enzymes tryptase and chymase, and MC_T_ that contain tryptase but not chymase [1]. MC_T_ are typically found in the mucosa, whereas MC_TC_ are more prominent in the connective tissue.

MC rely on their c-KIT receptor for proliferation and survival. This c-KIT is activated by its ligand stem cell factor (SCF) [3]. Furthermore, stimulation of c-KIT enhances MC activation, IL-6 secretion and MC adhesion [4]. It is hypothesized that the expression of SCF, for instance by the vascular endothelium, is an important chemotactic factor for MC progenitors.

MC are best known for their granules that contain infamous mediators such as histamine and tryptase, but they are capable of producing many more different substances such as leukotrienes, prostaglandins, growth factors and inflammatory cytokines [5]. Some of these, e.g., chymase and tryptase, are stored to be readily released after their activation. Other MC mediators have to be produced upon activation, for instance eicosanoids or cytokines, which can take up to 24 h [5]. The type of mediators that are produced is dependent upon the type of stimulus.

MC can be activated through a wide range of stimuli. The most well-known route of MC activation is through a crosslinking of IgE that is bound to the IgE-receptor. There are two IgE receptors: FcεRI, which has a tetrameric structure and a high affinity to IgE, and FcεRII, which generally is monomeric, and has a low affinity to IgE [6]. Other stimuli for MC activation are complement factors 3a and 5a, via toll-like receptors, IgG and cytokine receptors [4]. MC are also able to activate themselves in an autocrine manner, for instance through IL-5 and tryptase. A unique receptor to MC is the Mas-related G-protein receptor X2 (MRGPRX2). This receptor can be activated by many different ligands such as substance P, different small molecule drugs including neuromuscular blockers and antibiotics, and the components of snake and wasp venom [7]. The response of MC varies according to the type of stimulus, and mainly consists of a combination of degranulation, cytokine production and/or the synthesis of lipid mediators [8].

Although MC-deficient mice do not have decreased survival, MC probably have been an important asset of innate immunity in an evolutionary sense. MC probably originated >500 million years ago, and had a similar phenotype and function as the human cutaneous MC do nowadays, as elegantly shown by Wong et al. [9]. As part of the host defense, and due to its position in areas of the body that have a barrier function to the outside world, MC play a role in immunity against parasites, but also against bacterial and viral infections. Moreover, the proteases that they release upon activation can degrade the toxic components of those venoms of snakes and insects [10]. Next to these useful features of MC, they are most infamous for their role in allergic diseases. Furthermore, MC have been shown to contribute to the development of a number of cardiovascular diseases [11]. The increased prevalence of cardiovascular disease in mastocytosis supports the hypothesis that MC are important players in atherosclerosis, although the exact mechanism is not yet elucidated [12]. In this review, we will summarize the current knowledge regarding mast cell effects on atherosclerosis and heart diseases.

## 2. Mast Cells in Atherosclerosis

As early as 1954, MC were found in human atherosclerotic plaques [13]. At that time however, the role of the MC in the development of atherosclerosis, if any, was unclear. A few decades later, the discovery that MC activation in a low density lipoprotein (LDL)-containing medium leads to the formation of foam cells from macrophages, sparks further interest in the role of MC in atherosclerosis, as these data suggest that these MC have pro-atherogenic effects [14,15]. In vitro studies using rat MC show furthermore that mast cell granules are able to degrade high density lipoprotein (HDL)-particles, thereby inhibiting reverse cholesterol transport, a protective mechanism in atherosclerosis development [16]. At that time, pathology studies of human coronary arteries reveals that MC are not only present in atherosclerotic plaques, but that their numbers are significantly elevated in ruptured plaques, as compared with non-ruptured plaques [17]. Particularly in the adventitia, MC numbers are shown to increase upon plaque progression, and to be specifically high in plaques that rupture and show signs of thrombosis [18]. In the advanced atherosclerotic plaque, MC are shown to predominantly accumulate in the rupture-prone shoulder region, where these cells can affect the stability of the fibrous cap [19]. In human plaques, all MC contain tryptase, as demonstrated by immunohistochemical staining, and a subset contains chymase as well [20].

More recently, MC numbers are determined in human carotid endarterectomy plaques, which confirms the accumulation of MC during plaque progression [21]. MC numbers are actually increased in plaques which contain intraplaque hemorrhages, an important feature of plaque instability, suggesting that MC may contribute to the incidence of these hemorrhages. To support this hypothesis, the authors show that MC colocalizes with small intraplaque neovessels, which are prone to become leaky, as is demonstrated previously in coronary artery plaques as well [22]. MC numbers are also associated with neovessel density in the plaque, which indicates that MC-derived growth factors could induce neovascularization. For example, the MC-derived vascular endothelial growth factor (VEGF) and the basic fibroblast growth factor (bFGF) are associated with neovessel growth in tissue [23,24]. Interestingly, in the carotid endarterectomy study, MC numbers in the plaque are associated with the incidence of future cardiovascular events. In addition, circulating tryptase levels are elevated in patients that suffer from a cardiovascular event during a follow-up of the study, as compared to the patients that remain free of these cardiovascular events. Together, these data suggest that the MC may be actively involved in the destabilization of plaques in cardiovascular patients. These data are mostly based on histology, using a tryptase staining to identify MC in a 2-dimensional tissue section of the culprit plaque area. Although this approach gives highly relevant information regarding MC location in the plaque, it does not provide data regarding MC numbers or their activation status in the whole plaque. Very recently, a novel strategy to identify MC in human endarterectomy tissue was published, in which whole plaque tissue was enzymatically digested for flow cytometry analysis [25]. With this technique using CD45, CD117 and the FcεRI as markers, a distinct MC population was identified, and specific subsets were distinguished that had for example IgE bound on their surface, which were activated as measured by CD63 staining. The advantage of using this technique is the possibility of applying multiple markers to identify subsets of cells, which is not possible to such an extent using conventional immunohistochemical analysis. Importantly, this study also shows that not all MC in the plaque did stain positive for tryptase, which might be either due to recent degranulation, or to the fact that not all MC in the plaque contain tryptase. Thus, using only tryptase as a marker for MC in immunohistochemistry might result in an underestimation of the MC numbers. Taken together, these studies using human plaque tissue show that MC are present in human atherosclerotic plaque, where these cells seem to contribute to disease progression. However, causality is not proven, since intervention studies have up to date not been performed, but there are quite a number of experimental studies that firmly establish that MC promote atherogenesis. 

The first studies investigating the contribution of MCs to the development of atherosclerosis were published in 2007. On the one hand, MC deficiency in LDLr^−/−^ mice results in reduced atherosclerosis compared to MC-competent LDLr^−/−^ mice [26]. Repopulation with MC completely reestablishes atherogenesis, instigated by the MC-derived cytokines IFNγ and Interleukin (IL)-6. MC activation during atherogenesis on the other hand results in increased atherosclerotic lesion development [27]. In the same study, MC activation in the carotid artery of apoE^−/−^ mice leads to an increased incidence of intraplaque hemorrhage, an important feature of plaque instability, which could be completely prevented by treatment with the MC stabilizer cromolyn [27]. MC activation also results in an increase in leukocyte recruitment to the plaque, and additional follow-up studies clearly identify the neutrophil to be one of the major leukocytes to be recruited to the plaque in response to MC-derived CXCL1 (IL-8) [28]. Furthermore, MC activation can lead to an upregulation of adhesion molecules by the endothelial cells, thereby facilitating the adhesion and subsequent transmigration of leukocytes into the plaque, thus promoting plaque inflammation [29]. MC can also induce apoptosis of cells in the plaque such as macrophages [27], as well as endothelial cells [30,31] and smooth muscle cells [32,33], which may cause plaque erosion or rupture due to loss of cap stability. Particularly, MC-derived chymase is thought to be responsible for these pro-apoptotic effects of mast cells, as was demonstrated in an in vivo model, where a TLR4-mediated mast cell activation and subsequent plaque destabilization is proposed to be mediated at least partly via chymase [34]. 

Chymase inhibition in apoE^−/−^ mice results in plaque stabilization by increasing collagen content and reducing necrotic core size, suggesting that chymase inhibition is of therapeutic interest to prevent plaque instability [35]. Besides chymase, tryptase activity also contributes to plaque destabilization, as tryptase overexpression results in intraplaque hemorrhage [36]. 

For therapeutic intervention, the elucidation of MC activation mechanisms has been an important topic of research. Since the IgE-FcεRI pathway represents the main route of MC activation in allergic disease, its role in atherosclerosis has gained increasing attention. Enhanced IgE levels are associated with hyperlipidemia and cardiovascular diseases [37,38], suggesting that this is a relevant mechanism of MC activation in the atherosclerotic plaque. Indeed, episodes of systemic IgE-mediated MC activation during the development of atherosclerosis results in increased MC activation in the myocardium and a concomitant increase in atherosclerotic plaque size in a hyperlipidemic mouse model [28]. As expected, an inhibition of IgE by means of an anti-IgE antibody treatment reduces mast cell activation and plaque progression [39]. The relevance for human atherosclerosis is just recently established, as 40% of human plaque MC has IgE bound to their surface while expressing the activation marker CD63 [25]. The exact antigens of these IgEs however remain to be discovered. Furthermore, as Kritikou et al. show that a significant proportion of activated intraplaque MC does not have IgE bound to their surface [25], suggesting that other MC activation pathways may be involved in atherosclerosis as well, that remains to be discovered.

**Figure 1:**
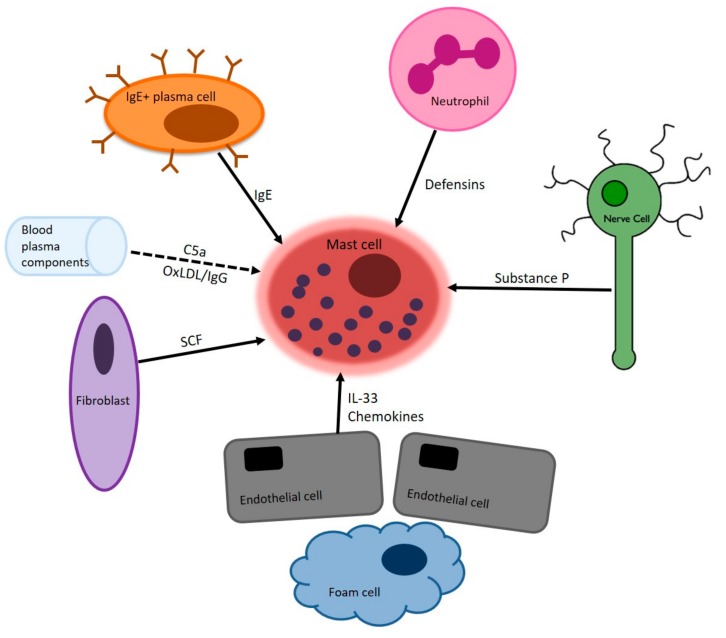
The mast cell is activated by various signals that arise from cells that are present in and around the atherosclerotic plaque. The mast cell is a tissue resident cell, and can adjust its actions according to stimuli from its environment. Here, the most important cells in the vascular wall and the atherosclerotic plaque are depicted. Each cell can deliver a different stimulus to cause mast cell activation. Subsequently, the activated mast cell produces different mediators which further enhance local inflammation and plaque instability (see Figure 2). SCF: Stem cell factor, C5a: Complement factor 5a, OxLDL/IgG: Oxidated LDL/IgG complexes (where LDL refers to low density lipoprotein).

**Figure 2:**
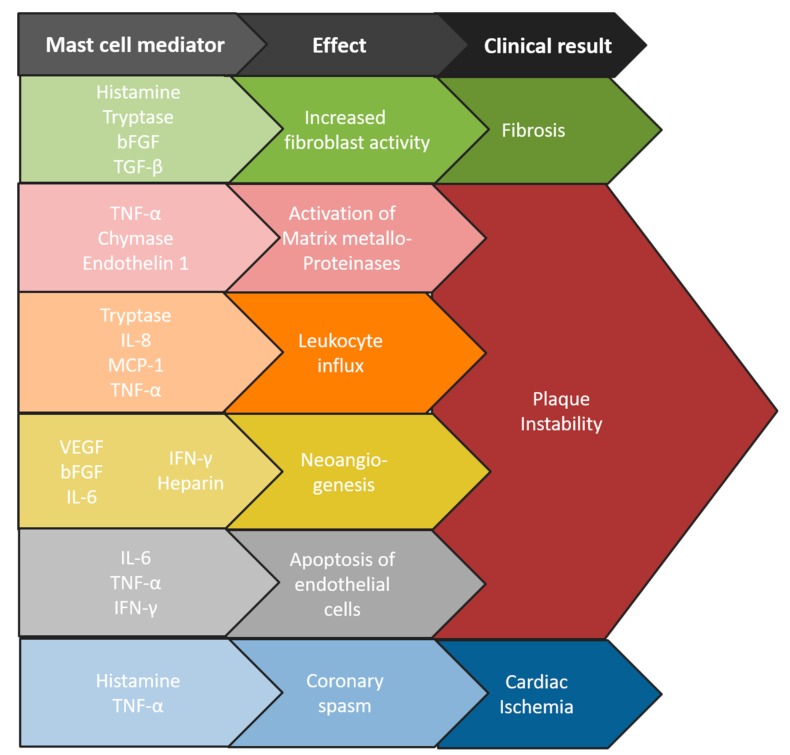
Mast cells (MC) mediators promote different aspects of cardiovascular disease. Upon activational signals from their environment, mast cells produce different kinds of mediators, which in their turn have pro-inflammatory or pro-fibrotic effects on the blood vessel wall and atherosclerotic plaque. For an explanation on the different substances, please see the text of the manuscript.

In summary, experimental studies clearly identify MC as pro-atherogenic immune cells that affect plaque development and progression by enhancing leukocyte influx, inducing the apoptosis of plaque cells and by promoting intraplaque hemorrhage. These data can, at least partly, explain the MC-associated effects observable in human atherosclerotic plaques and render MC as a promising therapeutic target in preventing atherosclerotic plaque destabilization. Figure 1 summarized the main stimuli that are present in the micro-environment of the atherosclerotic plaque. Figure 2 shows how MC mediators subsequently cause various aspects of cardiovascular disease.

## 3. Mast Cells in Cardiac Disease

A normal heart contains a relatively low number of MC within its myocardium [40]. However, the density of myocardial MC increases up to 6-fold when the heart is put under stress. For instance, increased cardiac MC numbers are found in an ischemia-reperfusion model in dogs. MC accumulation is most notable in areas with collagen deposition [41]. The same researchers provide evidence suggesting MC degranulation, and more specifically the release of histamine and TNF-α, after the induction of cardiac ischemia [42].

An interesting animal model to prove the pivotal role of MC in cardiac fibrosis is the c-KIT deficient W/Wv mouse model, in which mast cells fail to develop, and this therefore leads to MC deficiency. In an ischemia-reperfusion study in these MC deficient mice, more viable myocardial tissue was found after the ischemic event than in wild-type mice [43].

The association between MC and fibrosis has long been established. Key MC mediators in the induction of cardiac fibrosis are histamine, transforming growth factor β (TGF-β), several matrix metalloproteinases and MC-derived renin [44]. Interestingly, a large prospective observational cohort study shows that the use of histamine receptor-2 antagonists is associated with a 62% decreased risk of heart failure, after an adjustment for the obvious confounders [45].

While MC are important in the induction of pro-fibrotic processes, they can vice versa be activated by the mechanical stress that results from fibrosis of the tissue. This has not been proven directly for cardiac MC, but it appears that cardiac MC in rats are more susceptible to mechanical stress than for instance peritoneal or pleural MC [40]. Indirect evidence for the effect of mechanical stress on MC comes from studies in pulmonary fibrosis: Due to the fibrosis, the compliancy of pulmonary tissue decreases, occasioning shear stress to the resident MC, causing them to initiate TGF-β production [46,47].

An interesting although indirect form of evidence for the role of mast cell-derived histamine in cardiovascular disease derives from a study published in 2004 in which post-myocardial infarct patients are randomized for treatment with either the histamine 1 receptor antagonist loratidine or a placebo. Patients on the loratidine had a significant improvement of ischemic parameters induced by exercise testing two and three weeks after their infarction. 

In mice, activation of the histamine 2 receptor results in increased ischemia-reperfusion injury, suggesting that histamine aggravates ischemia in the heart via this receptor [48].

In short, the MC are resident cells of the healthy heart, and several studies have shown an increase in MC numbers post-ischemia. Furthermore, MC-derived substances, in particular histamine, appear to promote fibrosis in the process of cardiac remodeling.

## 4. Kounis Syndrome

In addition to atherosclerosis and fibrosis, MC can contribute to cardiac disease in a third way. Kounis syndrome was described as such in 1991, but the first reports of cardiac ischemia due to anaphylaxis were published decades earlier [49]. However, since 1991, acute coronary syndrome due to MC degranulation is termed Kounis syndrome. Kounis syndrome is caused by coronary spasms and/or the rupture of pre-existent atherosclerotic plaques due to MC activation [50]. The acute coronary syndrome occurs within one hour after exposition to the stimulus, and it can present as chest pain, ST-changes on the electrocardiogram, or cardiac arrhythmias [50]. To date, three types of Kounis syndrome have been described. Type 1, the most common subtype, is characterized by coronary spasms without any evidence of coronary atherosclerotic plaques. Type 2 results from a combination of coronary spasms with a plaque rupture. Type 3 is the most rare subtype, and is defined by a coronary artery stent occlusion, due to MC activation [50].

The most common triggers for the Kounis syndrome are antibiotics, insect stings, or food allergy, and this can thus be classified as an IgE-mediated allergic response which probably acts via the FcεRI receptor. However, non-IgE-mediated MC activation, such as in systemic mastocytosis, can theoretically also cause Kounis syndrome. In a case series of 10 patients with Kounis syndrome, four were later diagnosed with mast cell disease [51]. Furthermore, the Kounis syndrome was reported in association with scromboid syndrome, which is considered to be histamine poisoning due to the consumption of decayed fish, and has a very similar symptomatology to anaphylaxis [52]. This illustrates the important role of histamine in causing coronary spasms. Next to histamine, many other MC mediators are implicated to play a role in causing acute coronary syndrome upon MC activation. Tryptase induces matrix metalloproteinases that disrupt atherosclerotic plaque stability by degrading collagen fibers. Chymase and cathepsin-D, which are released by mast cells upon activation, can convert angiotensin I to angiotensin II, which can subsequently cause vasospasms. 

Kounis syndrome therefore represents a specific syndrome in which MC activation directly leads to cardiac morbidity, albeit via another etiology than in atherosclerosis or fibrosis.

## 5. Mastocytosis and Cardiovascular Morbidity

Mastocytosis is a clonal hematological disease in which aberrant mast cells accumulate. In most patients, a specific activating mutation in c-KIT is found. This D816V mutation causes an autonomic activity of the c-KIT receptor without its ligand stem cell factor, thereby causing increased MC proliferation and survival [53]. Mastocytosis is a disease of all ages, although in children it is mostly restricted to the skin, e.g., cutaneous mastocytosis, whereas most adults have systemic mastocytosis. According to the WHO criteria, systemic mastocytosis is defined by the presence of increased numbers of aberrant MC in at least one extracutaneous organ, most often the bone marrow [54].

Patients with mastocytosis can suffer from MC mediator-related symptoms such as pruritus, flushing, pyrosis, or anaphylaxis. In aggressive subtypes of systemic mastocytosis, MC infiltration of organs may also lead to organ dysfunction, like hepatic dysfunction, pleural effusion, or bone fractures [55]. Elevated levels of pro-inflammatory cytokines are detectable in patients with mastocytosis, probably explaining the constitutional symptoms that are often present [56].

Two previous studies show that the prevalence of cardiovascular disease is increased among patients with mastocytosis, however data on this subject are scarce [12,57]. In a large population-based cohort study from Denmark, an increased risk of myocardial infarction for patients with mastocytosis was found compared to a general control population (Table 1). Furthermore, the risk of a stroke was significantly higher among patients with mastocytosis as compared with controls [57].

Moreover, the prevalence of myocardial infarction is almost four times higher in patients with advanced subtypes of systemic mastocytosis, compared with indolent systemic mastocytosis, but no difference in a risk of stroke between the subtypes of systemic mastocytosis was observed [57]. 

Another case-control study measures carotid intima-media thickness and plasma lipids among 50 patients with systemic mastocytosis and 50 sex- and age-matched controls. In that study, a significantly increased prevalence of cardiovascular disease is demonstrated among the patients with mastocytosis when compared with the controls (20% versus 6%, respectively) [12]. There is a statistical tendency towards more plaques at the carotid intima-media thickness measurement for patients with mastocytosis, but this is not statistically significant. Surprisingly, patients with mastocytosis have lower mean levels of total cholesterol and LDL-cholesterol than the controls. The LDL levels are not correlated to serum tryptase levels [12]. Current cardiovascular prevention guidelines do not remark on the increased risk of cardiovascular disease in this patient population, probably because of its rarity [58]. However, in view of these findings it can be questioned whether these patients should receive intensified cardiovascular risk management. 

Non-mast cell clonal hematopoiesis is also associated with an increased risk of cardiovascular disease: In a large case-control study, the presence of one or more mutations without overt hematological disease leads to a doubled risk of coronary heart disease [59]. The authors propose that these mutations may alter macrophage biology and thereby stimulate atherosclerosis. It is unclear to which extent these findings and hypothesis can be extrapolated to mastocytosis.

## 6. Allergic Disease and Cardiovascular Morbidity

Whereas mastocytosis is a clonal disease of the mast cells, allergic diseases are characterized by an increased activity of ‘normal’ mast cells via their activation through IgE. Accumulating evidence suggests that patients with allergies are also at increased risk of atherosclerosis. This is not surprising, regarding the above-mentioned evidence suggesting a role for IgE in atherosclerosis. 

Asthma for instance is an exemplary disease in which both IgE-driven mast cell activation and more chronic Th2 activity leads to an inflammation of the airways. Furthermore, high amounts of macrophages and eosinophils can be found in the broncheoalveolar lavage fluid of asthmatic patients [60]. Considering the fact that these same cells are found in atherosclerotic plaques, it is not surprising that allergic asthma is associated with atherosclerosis. The available evidence on cardiovascular morbidity and asthma was nicely summarized by Liu et al. in 2016 [61]. Cohort studies revealed an odd’s ratios between 1.4 and 3.8 for atherosclerotic disease in patients with allergic rhinitis and/or asthma, when corrected for sociodemographic factors and comorbidities [62,63,64]. In one American case-control study, adults with asthma also had a 3.28-fold risk of heart failure. However, comorbid allergies are not clearly associated with cardiovascular disease when analyzed separately from asthma [64].

One interesting study followed children from birth until the age of five years, and measures carotid intima-media thickness (CIMT) and arterial wall stiffness. They found that children with allergies and atopic parents had a significantly larger CIMT compared with children without allergies of parental allergies [65]. This shows that the process of atherosclerosis may start at young age in atopic individuals. Koskinen et al. however found that CIMT is not predictive of cardiovascular disease before the age of eight years [66]. Moreover, another recent study found no difference in the prevalence of atherosclerosis between patients with or without asthma [67]. More data from other cohort studies support this finding [61]. The evidence on lipid levels among atopic individuals is also conflicting, and is highly variable between cohorts. Overall, it appears that increased levels of total cholesterol and non-HDL levels are associated with allergic disease in Asian and Hispanic people, whereas an inverse relationship is seen among people of Western European or African descent [68].

**Table 1:** Clinical data on the risk of cardiovascular disease associated with mastocytosis or allergic diseases.

Cohort	Primary Disease	Comorbidity	Hazard Ratio (95% CI)
Danish population registry [57]	Mastocytosis	Stroke	1.6 (1.13–2.27)
		Myocardial infarction	1.4 (0.9–2.3)
Dutch case-control study [12]	Mastocytosis	Stroke	5.0 (0.6–41.3)
		Coronary heart disease	2.5 (0.5–12.3)
Italian population survey [62]	Allergic rhinitis, asthma, or both	Atherosclerosis †	3.9 (1.3–11.5)
Austrian adolescents [62]	Allergic rhinitis, asthma, or both	Atherosclerosis †	3.0 (1.1–7.9)
USA cohort [63]	Allergic rhinitis	Coronary heart disease	1.40 (1.02–1.92)
	Wheezing	Coronary heart disease	2.64 (1.79–3.9)
USA case-matched cohort [64]	Asthma	Coronary heart disease	1.40 (1.35–1.45)
		Stroke	1.20 (1.15–1.25)
		Heart failure	2.14 (2.06–2.22)
Polish case-control study [67]	Asthma	Atherosclerotic plaque in LCCA ‡	1.2 (0.55–0.91)
		Atherosclerotic plaque in RCCA ‡	0.31 (0.10–0.91)

† The presence of atherosclerosis is determined by carotid artery ultrasonography. ‡ LCCA = left common carotid artery; RCCA = right common carotid artery. Carotid intima media thickness was not different for asthma patients compared to controls.

It can be questioned whether CIMT is a reliable surrogate for cardiovascular disease: Mere measurement of plaque thickness appears to be of limited value, but when CIMT is also used to collect data on the plaque number, plaque vascularization etc., it can certainly be useful to predict cardiovascular morbidity [69].

Another possible explanation for the different findings between these studies is the correction for possible confounders: Since atherosclerosis is influenced by so many factors, it is virtually impossible to compare groups in the observational, often retrospective, cohort design that most studies have used. For instance, most patients with asthma use corticosteroids in some shape or form, and it is well known that corticosteroid excess is associated with cardiovascular disease [70]. Moreover, the racial predisposition for both allergies and atherosclerosis is not taken into account in most studies. Furthermore, patients with asthma and atopic dermatitis appear to be ‘unhealthy’: An increased presence of obesity and nicotine abuse in this patient category has been found [71,72,73]. Interestingly, it appears that MC are able to maintain a low-grade inflammatory state within adipose tissue and are vice versa activated by adipocytes, rendering these MC as a link between obesity and asthma.

In conclusion, there is no unequivocal proof that allergic disease is associated with increased cardiovascular morbidity, however, individuals with allergic disease may gather multiple risk factors for cardiovascular disease including a chronic state of inflammation, increased CIMT, obesity and smoking. 

## 7. Conclusions

There is a wide body of evidence suggesting a pivotal role for MC in different aspects of cardiovascular disease. MC are abundant in atherosclerotic plaques. Furthermore, different diseases in which MC play a key role are associated with an risk of atherosclerosis. This represents an interesting target for therapy. Simple drugs such as antihistamines may form a very feasible addition to the standard regime for the (secondary) prevention of atherosclerosis. We believe that in addition to the now extensive work that has already been done in vitro and in animal studies, it is now time for intervention studies in humans to study the effect of targeting MC for atherosclerosis.
